# Supplementary material for: Causal Relationship between Aging and Anorexia Nervosa: A White-Matter-Microstructure-Mediated Mendelian Randomization Analysis
Source: Biomedicines. 2024 Aug 16;12(8):1874. doi: 10.3390/biomedicines12081874 (PMC11351342; doi:10.3390/biomedicines12081874)
Supplement: Supplementary file 1 [file biomedicines-12-01874-s001.zip › Supplementary Figure/Figure S4/Singe SNP plot of MR analysis for TL to ubm-b-2079.pdf]

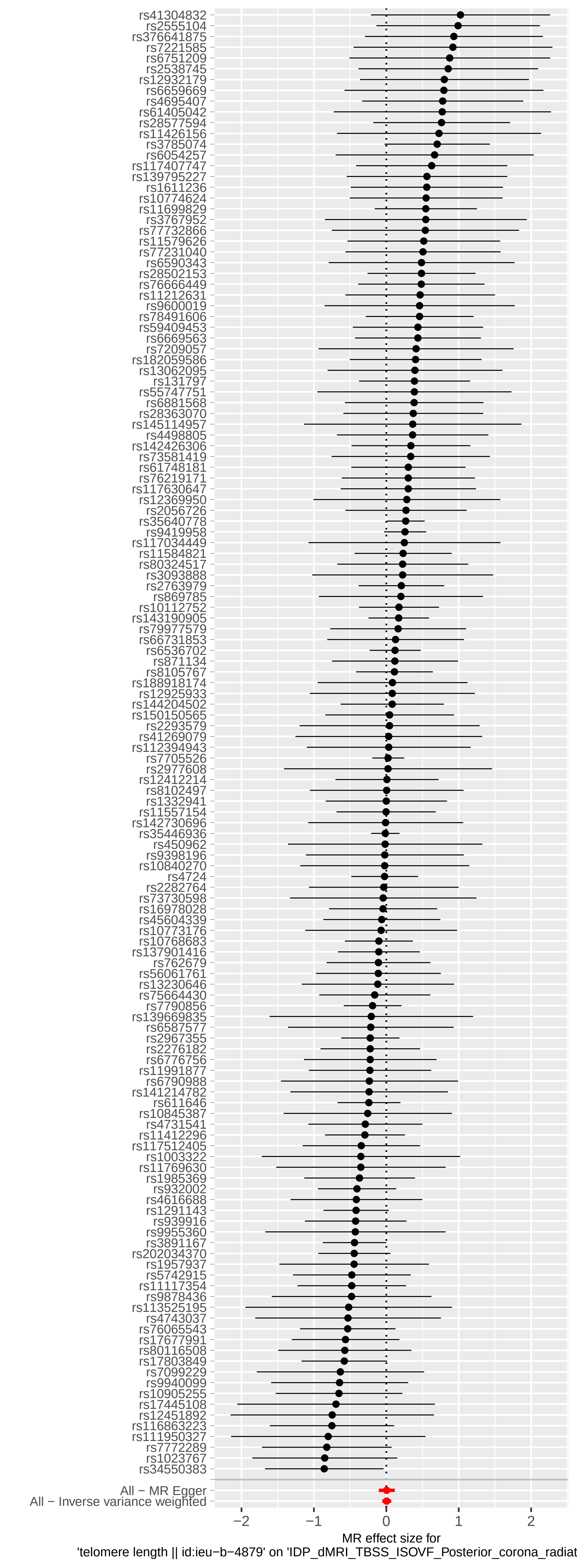
rs41304832
rs2555104
rs376641875
rs7221585
rs6751209
rs2538745
rs12932179
rs6659669
rs4695407
rs61405042
rs28577594
rs11426156
rs3785074
rs6054257
rs117407747
rs139795227
rs1611236
rs10774624
rs11699829
rs3767952
rs77732866
rs11579626
rs77231040
rs6590343
rs28502153
rs76666449
rs11212631
rs9600019
rs78491606
rs59409453
rs6669563
rs7209057
rs182059586
rs13062095
rs131797
rs55747751
rs6881568
rs28363070
rs145114957
rs4498805
rs142426306
rs73581419
rs61748181
rs76219171
rs117630647
rs12369950
rs2056726
rs35640778
rs9419958
rs117034449
rs11584821
rs80324517
rs3093888
rs2763979
rs869785
rs10112752
rs143190905
rs79977579
rs66731853
rs6536702
rs871134
rs8105767
rs188918174
rs12925933
rs144204502
rs150150565
rs2293579
rs41269079
rs112394943
rs7705526
rs2977608
rs12412214
rs8102497
rs1332941
rs11557154
rs142730696
rs35446936
rs450962
rs9398196
rs10840270
rs4724
rs2282764
rs73730598
rs16978028
rs45604339
rs10773176
rs10768683
rs137901416
rs762679
rs56061761
rs13230646
rs75664430
rs7790856
rs139669835
rs6587577
rs2967355
rs2276182
rs6776756
rs11991877
rs6790988
rs141214782
rs611646
rs10845387
rs4731541
rs11412296
rs117512405
rs1003322
rs11769630
rs1985369
rs932002
rs4616688
rs1291143
rs939916
rs9955360
rs3891167
rs202034370
rs1957937
rs5742915
rs11117354
rs9878436
rs113525195
rs4743037
rs76065543
rs17677991
rs80116508
rs17803849
rs7099229
rs9940099
rs10905255
rs17445108
rs12451892
rs116863223
rs111950327
rs7772289
rs1023767
rs34550383
All − MR Egger
All − Inverse variance weighted
−2
−1
0
1
2
MR effect size for
'telomere length || id:ieu−b−4879' on 'IDP_dMRI_TBSS_ISOVF_Posterior_corona_radiat
